# Case report: liver failure as a debut of autoimmune hepatitis triggered by dengue virus in a pregnant woman

**DOI:** 10.17843/rpmesp.2024.412.13485

**Published:** 2024-06-13

**Authors:** Karina Sato-Espinoza, Diego Berrospi, Javier Diaz-Ferrer

**Affiliations:** 1 Escuela de Medicina. Universidad Peruana de Ciencias Aplicadas (UPC), Lima, Peru. Universidad Peruana de Ciencias Aplicadas Escuela de Medicina Universidad Peruana de Ciencias Aplicadas (UPC) Lima Peru; 2 Facultad de Medicina, Universidad Peruana Cayetano Heredia (UPCH), Lima, Peru. Universidad Peruana Cayetano Heredia Facultad de Medicina Universidad Peruana Cayetano Heredia (UPCH) Lima Peru; 3 Hospital Nacional Edgardo Rebagliati Martins, Lima, Peru. Hospital Nacional Edgardo Rebagliati Martins Lima Peru; 4 Facultad de Medicina, Universidad San Martín de Porres, Lima, Peru. Universidad de San Martín de Porres Facultad de Medicina Universidad San Martín de Porres Lima Peru

**Keywords:** Hepatitis, autoimmune, dengue virus, pregnant, liver failure, acute, infections

## Abstract

Autoimmune hepatitis (AIH) is a complex condition with unclear origins, involving genetic susceptibility and environmental triggers that lead to immune system dysfunction. We report a case of a pregnant woman from a mosquito-borne disease-endemic area who presented jaundice, abdominal pain, and pruritus, complicated by acute liver failure. Immunological markers showed AIH triggered by dengue virus infection, which was confirmed by a positive IgM test. Treatment with supportive care followed by steroids and azathioprine resulted in favorable outcomes, averting the need for a liver transplant. Although AIH can be triggered by viruses, the role of dengue in its pathogenesis remains poorly understood. Regular clinical monitoring is vital for managing AIH, particularly during pregnancy, due to variable immune status and treatment responses. Further research is necessary to understand the link between dengue infection and AIH. Individualized treatment strategies are crucial, especially during pregnancy, in order to ensure favorable outcomes.

## INTRODUCTION

Dengue is endemic in several regions of Peru, particularly in the northern regions and the Amazon, where high morbidity and mortality rates are reported each year ^1^^,^^2^. The main vector of transmission is the *Aedes aegypti* mosquito. Efforts are currently underway to improve public education and public awareness regarding dengue prevention ^3^. However, dengue remains a public health challenge worldwide. In 2023, the World Health Organization (WHO) ^4^ reported, in the Americas region, which includes Peru, Brazil, Mexico, and Colombia, a total of 4.1 million suspected dengue cases, 6710 severe cases, and 2049 deaths. The African regions accumulated 171,991 dengue cases and 753 deaths; the Southeast Asia region accumulated 308,167 dengue cases and 1745 deaths. Other regions, such as the Eastern Mediterranean, Europe, and the Western Pacific, also face dengue morbidity and mortality. Currently, dengue is a persistent public health challenge due to periodic epidemiological outbreaks, putting great pressure on health systems due to its high infection rates.

Dengue can have a variety of symptoms, with fever and skin lesions being the most common ^5^. However, it can also affect the digestive system, causing symptoms such as abdominal pain, jaundice, hepatitis and, in severe cases, acute liver failure ^6^. Distinguishing the symptoms of dengue and autoimmune hepatitis (AIH) is crucial, because they may overlap. Following dengue infection, the immune system may generate autoreactive T cells capable of reacting to liver antigens, causing an abnormal autoimmune response. However, unlike AIH, specific biomarkers such as ANA, LKM-1 and ASMA are not typically found in dengue infection. We report the first case in Peru and the second worldwide ^7^ of a patient who debuted with AIH and acute liver failure, triggered by dengue virus infection.

Understanding the symptoms, diagnosis and treatment of mosquito-borne infections is essential. However, it is challenging to diagnose cases like our patient. Therefore, it is crucial to raise differential diagnoses when the clinical presentation does not follow the typical pattern. Existing literature highlights the potential risk of developing immunological diseases as a result of viral infections ^8^^,^^9^. In addition, IAH is a complex condition characterized by unclear mechanisms involving the interaction between genetic components and environmental triggers. This complex condition emphasizes the importance of correct diagnosis and treatment to prevent future complications ^10^.

## CASE REPORT

### Patient information

We present the case of a 34-year-old woman, 26 weeks pregnant, from the city of Iquitos, in the Amazon region of Peru. She went to the emergency room of the Regional Hospital of Loreto, with a persistent condition of two weeks, characterized by reduced appetite, abdominal pain, jaundice, pruritus and headache. There was no significant history and she did not present any complications during her gestation.

### Clinical findings

Physical examination showed jaundice of the skin and mucous membranes and pain in the right hypochondrium on palpation. Gynecological examination showed a uterine height of 22 cm and a fetal heart rate of 150 beats per minute, accompanied by fetal movements.

### Timeline

Laboratory tests confirmed the presence of IgM antibodies and the absence of IgG antibodies, indicating active dengue virus infection (Table 1). The diagnosis was confirmed by the hospital’s infectious disease service. During this period, the DENV-2 serotype was the most prevalent in the city of Iquitos. The patient was diagnosed with hepatitis caused by dengue virus and transferred to a tertiary level hospital Edgardo Rebagliati Martins in Lima, to be treated in the Hepatology Service.

**Table 1:** Test results**.**

Test	Results
Dengue virus panel	NS1=negative IgM=Positive (active infection) IgG=negative
Thick blood smear for malaria	Negative
Leptospira (IgG, IgM)	Both negatives
Cytomegalovirus	IgG=Positive (previous infection) IgM=Negative
Coagulation	aPTT=23 s TP=13.6 s
Liver function	TBIL=19.46 mg/dL DB=9.63 mg/dL IB=9.83 mg/dL AST=2119 mg/dL ALT=1506 mg/dL AP=119 mg/dL Albumin= 2.32 mg/dL
Serology for viral hepatitis	HBsAg=Negative Anti-HBc=Negative Anti-HCV=Negative
Renal function	Creatinine=0.42 mg/dL Urea=11 mg/dL
Complete blood count	White blood cells=4900 Hemoglobin=12 mg/dL Platelet count=209,000
Antibodies	IgG=2544 (6-16) IgA=396 (80-350) IgM=206 (37-286)
ANA (antinuclear antibody)	1:60 (speckled pattern)
LKM-1 (Liver kidney microsomal antibody type 1)	3.3
ASMA (anti-smooth muscle antibody)	<1:20

NS1: non-structural protein1, Ig: Immunoglobulin, aPTT: activated partial thromboplastin time, PT: prothrombin time, TBIL: Total bilirubin, DB: direct bilirubin, IB: indirect bilirubin, AST: aspartate aminotransferase, ALT: alanine aminotransferase, AP: alkaline phosphate, mg/dL: milligrams per deciliter, HBsAg: hepatitis B surface antigen, anti-HBc: Hepatitis B core antibody, anti-HCV: Hepatitis C antibody.

### Diagnostic evaluation

After one month of persistent symptoms, when she was in the Hepatology Service, her clinical condition worsened, presenting alteration of the sensorium and acute liver failure. The King’s College criteria ^11^ were applied, and the patient met the criteria for acute liver failure, so she was admitted to the liver transplant list. Due to her deteriorating condition, she was transferred to the intensive care unit (ICU) for strict monitoring.

### Therapeutic intervention

**Figure 1:**
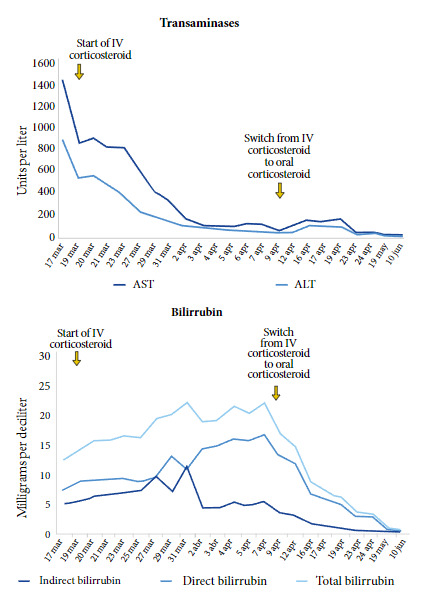
Liver function results.

It was suspected that the acute liver failure could be a consequence of AIH, triggered by acute dengue virus infection. Immunological markers were dosed and found to be increased, confirming the suspicion. Treatment started with methylprednisolone 50 mg per day. The response to treatment was positive at clinical and laboratory level (Figure 1). Due to the substantial improvement of the patient, it was decided to remove her from the transplant list. However, the pregnancy was complicated with premature rupture of membranes and acute fetal distress, requiring an emergency cesarean section at 29 weeks gestation. The procedure resulted in a healthy, uncomplicated newborn with an adequate Apgar score. But due to prematurity, she remained four weeks in the neonatal intensive care unit, showing a stable and promising recovery. The patient continued receiving intravenous corticoids, and all her laboratory parameters stabilized, so it was decided to switch to oral treatment with an initial prednisone dose of 50 mg per day, which gradually decreased to 10 mg before discharge (Figure 1). All her laboratory and imaging controls were normal and no additional complications were found. The patient was discharged on a maintenance dose of prednisone 5 mg and azathioprine 50 mg per day.

### Follow-up and results

One month after her discharge, the patient returned for her follow-up appointment, she showed no symptoms and normal transaminase and bilirubin levels. She was instructed to continue treatment with prednisone and azathioprine. Furthermore, the newborn remained healthy and did not experience any additional complications.

## DISCUSSION

Autoimmune hepatitis (AIH) is a complex disease influenced by genetic, epigenetic, immunological and environmental factors. It predominantly affects women in a bimodal presentation ^12^. Although the exact mechanism remains unknown, it is hypothesized that the production of autoreactive T cells acts against self-tolerance to hepatic autoantigens. In addition, overexpression of Th1 and Th17 cells, which mediate the proinflammatory response, plays an important role in its pathogenesis ^13^. Some studies report that viral infections can trigger this immune response ^7^^,^^8^^,^^9^. However, information on dengue virus as a possible immune system trigger is limited, with only one case report associating dengue infection and AIH ^7^.

Serology for dengue IgM infection may show false positives in certain patients, as has been reported in systemic lupus erythematosus and *in vitro* studies ^15^^,^^16^. One hypothesis is that this phenomenon may be due to cross-reactivity between the nonstructural (NS) protein that is specific to dengue virus and its interaction with host cells, or it could be due to the presence of rheumatoid factor antibodies, which can trigger nonspecific binding in the coating on IgM anti-human antibodies ^16^^,^^17^. Our patient initially had nonspecific symptoms and mild elevation in liver function tests, which supported the finding of dengue virus as the primary infection that produced an activation of the immune system that subsequently triggered the onset of AIH, causing a further increase in liver function test values. Autoimmune tests were carried out due to the torpid clinical and laboratory course, revealing positive autoantibodies compatible with AIH in the patient. However, a liver biopsy to confirm the diagnosis was considered unfeasible due to gestation. The risk outweighed the benefits and there was a high probability for the diagnosis of AIH. A limitation of this case report was the absence of liver biopsy confirmation. However, the favorable response to corticosteroid therapy in our patient confirms the hypothesis that the presentation was attributable to an autoimmune response triggered by dengue virus infection, leading to acute liver failure. This occurrence is rare due to protective immunologic changes during pregnancy. Elevated estrogen and progesterone levels contribute to an increase in regulatory and Th2 T cells, involved in the anti-inflammatory pathway, while decreasing the expression of Th1 and Th17 cells, corresponding to the pro-inflammatory pathway ^17^. Corticosteroid therapy was effective and the patient was successfully removed from the liver transplant list. During the postpartum period, azathioprine was introduced along with continuous prednisone therapy to maintain disease control, given the high risk of flares due to decreased hormone levels, which restore the proinflammatory state of Th1 and Th17 cells ^18^. Close monitoring during the first six months postpartum is crucial to identify disease flares ^12^^,^^17^^,^^19^. 

The positive response to corticosteroid therapy confirmed our suspicion of AIH in a patient infected with dengue virus. It is crucial that patients who respond well initially to high-dose corticosteroids be considered for adjuvant therapy, such as azathioprine, while gradually reducing the corticosteroid dose to minimize side effects and maintain a good clinical and laboratory response ^12^^,^^17^. Regular follow-up is essential for at least a couple of years after diagnosis to maintain disease control and prevent relapses ^17^.

We present the first case of a pregnant woman with dengue virus infection who subsequently developed AIH, confirmed by clinical course, immunological markers and positive response to treatment with corticoids and azathioprine. It is important for regions with high prevalence of dengue infection and patients susceptible to autoimmune diseases to consider this as a potential differential diagnosis. The exact mechanism by which this virus triggers AIH in susceptible patients remains unclear and further studies are needed due to the high prevalence of mosquito-borne diseases worldwide.
