# Supplementary material for: Case report: liver failure as a debut of autoimmune hepatitis triggered by dengue virus in a pregnant woman
Source: Rev Peru Med Exp Salud Publica. 2024 Jun 13;41(2):209–13. doi: 10.17843/rpmesp.2024.412.13485 (PMC11300697; doi:10.17843/rpmesp.2024.412.13485)
Supplement: Translation

# Translation: Falla hepática aguda como debut de hepatitis autoinmune desencadenada por dengue en una gestante: reporte de caso

**Authors:** Karina Sato-Espinoza, Diego Berrospi, Javier Diaz-Ferrer

**Affiliations:** 1 Escuela de Medicina. Universidad Peruana de Ciencias Aplicadas (UPC), Lima, Perú.; 2 Facultad de Medicina. Universidad Peruana Cayetano Heredia (UPCH), Lima, Perú.; 3 Hospital Nacional Edgardo Rebagliati Martins, Lima, Perú.; 4 Facultad de Medicina. Universidad San Martin de Porres. Lima, Perú.

**Keywords:** Hepatitis Autoinmune, Virus del Dengue, Embarazo, Insuficiencia Hepática Aguda, Infecciones

## Abstract

La hepatitis autoinmune (HAI) es una condición compleja con etiología poco clara, que engloba componentes genéticos y ambientales que pueden desencadenar disfunción del sistema inmunológico. Presentamos el caso de una gestante de una zona endémica de dengue que se presentó con ictericia, dolor abdominal y prurito, complicándose con insuficiencia hepática aguda. Los marcadores inmunológicos indicaron HAI desencadenada por la infección del virus del dengue, confirmada por una prueba positiva de IgM. El tratamiento con cuidados de soporte seguido de corticoterapia y azatioprina tuvo resultados clínicos y laboratoriales favorables, evitando la necesidad de un trasplante de hígado. Diversos virus han sido descritos como desencadenantes de HAI; sin embargo, el papel del virus del dengue en su patogénesis sigue siendo desconocido. El monitoreo clínico regular es vital para el manejo de la HAI, especialmente durante el embarazo, debido a las alteraciones inmunológicas y las diversas respuestas al tratamiento. Se necesita más investigación para dilucidar el vínculo entre la infección por dengue y el desarrollo de HAI. El tratamiento individualizado en estos pacientes es crucial, especialmente durante el embarazo, para disminuir el riesgo de complicaciones.

## INTRODUCCIÓN

El dengue es endémico en varias regiones del Perú, particularmente en el norte y la Amazonía, donde se reportan altas tasas de morbilidad y mortalidad cada año ^1^^,^^2^. El principal vector de transmisión es el mosquito *Aedes aegypti*. Actualmente, se están realizando esfuerzos para mejorar la educación en la población y la concientización pública sobre la prevención del dengue ^3^. Sin embargo, el dengue continúa siendo un desafío para la salud pública en todo el mundo. En el 2023, la Organización Mundial de la Salud (OMS) ^4^ reportó que, en la región de América, que incluye Perú, Brasil, México y Colombia, se observó un total de 4,1 millones de casos sospechosos de dengue, 6710 casos graves y 2049 muertes. Las regiones de África acumularon 171 991 casos de dengue y 753 muertes; la región del sudeste asiático acumuló 308 167 casos de dengue y 1745 muertes. Otras regiones reportadas, como el Mediterráneo oriental, Europa y el Pacífico occidental, también enfrentan morbilidad y mortalidad por dengue. Actualmente, el dengue es un desafío persistente de salud pública debido a los brotes epidemiológicos periódicos, ejerciendo una gran presión en los sistemas de salud por sus altas tasas de infección.

El virus del dengue puede presentarse con diversos síntomas, siendo la fiebre y las lesiones cutáneas las más comunes ^5^. Sin embargo, también puede involucrar el sistema digestivo, causando síntomas como dolor abdominal, ictericia, hepatitis y, en casos graves, insuficiencia hepática aguda ^6^. Distinguir los síntomas del dengue y la hepatitis autoinmune (HAI) es crucial, debido a que puede existir una superposición. Tras una infección por dengue, el sistema inmunológico puede generar células T autorreactivas capaces de reaccionar a antígenos hepáticos, provocando una respuesta autoinmune anómala. Sin embargo, a diferencia de la HAI, los biomarcadores específicos como ANA, LKM-1 y ASMA no se encuentran típicamente en la infección por dengue. Reportamos el primer caso en Perú y el segundo a nivel mundial ^7^ de una paciente que debutó con HAI e insuficiencia hepática aguda, desencadenada por la infección por el virus del dengue.

Es esencial comprender los síntomas, diagnóstico y tratamiento de las infecciones transmitidas por mosquitos. Sin embargo, es un desafío diagnosticar casos como el de nuestra paciente. Por lo que es crucial plantear diagnósticos diferenciales cuando la presentación clínica no sigue el patrón típico. La literatura existente destaca el riesgo potencial de desarrollar enfermedades inmunológicas como resultado de infecciones virales ^8^^,^^9^. Además, la HAI es una condición compleja caracterizada por mecanismos poco claros que involucran la interacción entre componentes genéticos y desencadenantes ambientales. Esta patología compleja enfatiza la importancia de un correcto diagnóstico y tratamiento para prevenir futuras complicaciones ^10^.

## REPORTE DE CASO

### Información del paciente

Mujer de 34 años con 26 semanas de embarazo, procedente de la ciudad de Iquitos, en la región de la Amazonía del Perú. Acudió a la emergencia del Hospital Regional de Loreto, con un cuadro persistente de dos semanas, caracterizado por hiporexia, dolor abdominal, ictericia, prurito y cefalea. No se tuvo antecedentes significativos, y no presentó ninguna complicación durante su gestación.

### Hallazgos clínicos

Durante el examen físico, se observó ictericia de piel y mucosas, dolor en hipocondrio derecho a la palpación. En el examen ginecológico, se midió una altura uterina de 22 cm y una frecuencia cardíaca fetal de 150 latidos por minuto, acompañada de movimientos fetales.

### Línea de tiempo

Se realizaron pruebas de laboratorio que confirmaron la presencia de anticuerpos IgM y la ausencia de anticuerpos IgG, lo que indica una infección activa del virus del dengue (Tabla 1). El diagnóstico fue confirmado por el servicio de infectología del hospital. Durante este período, el serotipo DENV-2 era el más prevalente en la ciudad de Iquitos. La paciente fue diagnosticada con hepatitis causada por el virus del dengue y transferida a un hospital de tercer nivel Edgardo Rebagliati Martins en Lima, para ser atendida en el Servicio de Hepatología.

**Tabla 1:** Resultados laboratoriales.

Prueba	Resultados
Panel virus del dengue	NS1=negativo IgM=positivo (infección activa) IgG=negativo
Frotis gota gruesa para malaria	Negativo
Leptospira (IgG, IgM)	Ambos negativos
Citomegalovirus	IgG=positivo (infección previa) IgM=negativo
Coagulación	aPTT=23 s TP=13,6 s
Función hepática	TBIL=19,46 mg/dL BD=9,63 mg/dL BI=9,83 mg/dL AST=2119 mg/dL ALT=1506 mg/dL FA=119 mg/dL Albumina= 2,32 mg/dL
Serología de hepatitis viral	HBsAg=negativo Anti-HBc=negativo Anti-HCV=negativo
Función renal	Creatinina=0,42 mg/dL Urea=11 mg/dL
Hemograma	Leucocitos=4900 Hemoglobina=12 mg/dL Plaquetas=209 000
Anticuerpos	IgG=2544 (6-16) IgA=396 (80-350) IgM=206 (37-286)
ANA (anticuerpo antinucleares)	1:60 (patrón moteado)
LKM-1 (Anticuerpos anti microsomal hepático 1)	3,3
ASMA (anticuerpos antimúsculo liso)	<1:20

NS1: proteína no estructural 1, Ig: inmunoglobulina, aPTT: tiempo de tromboplastina parcial activada, PT: tiempo de protrombina, TBIL: bilirrubina total, BD: bilirrubina directa, BI: bilirrubina indirecta, AST: aspartato aminotransferasa, ALT: alanino aminotransferasa, FA: fosfatasa alcalina, HBsAg: antígeno de superficie hepatitis B, anti-HBc: anti-core hepatitis B, anti-HCV: anticuerpo hepatitis C.

### Evaluación diagnóstica

Después de un mes de síntomas persistentes, cuando se encontraba en el Servicio de Hepatología, su condición clínica empeoró, presentando alteración del sensorio e insuficiencia hepática aguda. Se evaluaron los criterios del King’s College ^11^, y la paciente cumplió con los criterios de insuficiencia hepática aguda, por lo que fue ingresada a la lista de trasplante hepático. Debido al deterioro de su condición, fue trasladada a la unidad de cuidados intensivos (UCI) para un monitoreo estricto.

### Intervención terapéutica

Se sospechó que la insuficiencia hepática aguda podría ser a consecuencia de la HAI, desencadenada por la infección aguda del virus del dengue. Se dosaron marcadores inmunológi­cos, que resultaron incrementados, lo que confirmó la sospecha. Se inició tratamiento con metilprednisolona 50 mg por día. La respuesta a la corticoterapia fue positiva a nivel clínico y laboratorial (Figura 1). Debido a la mejora sustancial de la paciente, se decidió su retiro de la lista de trasplante. Sin embargo, el embarazo se complicó con la ruptura prematura de membranas y sufrimiento fetal agudo, por lo que se requirió una cesárea de emergencia a las 29 semanas de gestación. El procedimiento resultó en un recién nacido sano, sin complicaciones, con un adecuado puntaje de Apgar. Pero debido a la prematuridad, permaneció cuatro semanas en la unidad de cuidados intensivos neonatales, mostrando una recuperación estable y prometedora. La paciente continuó recibiendo corticoterapia endovenosa, y todos sus parámetros de laboratorio se estabilizaron, por lo que se decidió cambiar a corticoterapia oral con una dosis inicial de prednisona de 50 mg por día, que disminuyó gradualmente hasta alcanzar 10 mg antes del alta médica (Figura 1). Todos sus controles de laboratorio y de imagen fueron normales y no se encontraron complicaciones adicionales. Se dio de alta a la paciente con una dosis de mantenimiento de prednisona de 5 mg y azatioprina 50 mg por día.

**Figura 1:** Resultados de la función hepática

### Seguimiento y resultados

Un mes después de su alta, la paciente regresó para su cita de control, mostrandose asintomática y con niveles de transaminasas y bilirrubina normales. Se le indicó continuar con el tratamiento de prednisona y azatioprina. Además, el recién nacido se mantuvo saludable y no experimentó ninguna complicación adicional.

## DISCUSIÓN

La hepatitis autoinmune (HAI) es una enfermedad compleja influenciada por factores genéticos, epigenéticos, inmunológicos y ambientales. Afecta predominantemente a las mujeres en una presentación bimodal ^12^. Si bien el mecanismo exacto sigue siendo desconocido, se tiene la hipótesis de que la producción de células T autorreactivas actúa en contra de la autotolerancia a los autoantígenos hepáticos. Además, la sobreexpresión de las células Th1 y Th17, que median una respuesta proinflamatoria, tiene un papel importante en su patogénesis ^13^^,^^14^. Algunos estudios reportan que las infecciones virales pueden desencadenar esta respuesta inmune ^7^^,^^8^^,^^9^. Sin embargo, la información sobre el virus del dengue como posible desencadenante del sistema inmunológico es limitada, con solo un reporte de caso que asocia infección por dengue y HAI ^7^.

La serología de la infección por IgM del dengue puede mostrar falsos positivos en ciertos pacientes, como se ha reportado en lupus eritematoso sistémico y en estudios *in vitro*^15^^,^^16^. Una hipótesis es que este fenómeno puede deberse a la reactividad cruzada entre la proteína no estructural (NS) que es específica del virus del dengue y su interacción con las células del huésped, o podría deberse a la presencia de anticuerpos del factor reumatoideo, que puede desencadenar una unión no específica en el recubrimiento en los anticuerpos anti-humanos IgM ^16^^,^^17^. Nuestra paciente inicialmente presentó síntomas inespecíficos y una leve elevación en las pruebas de función hepática, lo que respaldó el hallazgo del virus del dengue como la infección primaria que produjo una activación del sistema inmunológico que posteriormente desencadenó el inicio de la HAI, generando un aumento adicional en los valores de las pruebas de función hepática. Debido al curso tórpido clínico y laboratorial, se realizó un panel autoinmune, revelando autoanticuerpos positivos compatibles con HAI en nuestra gestante. Sin embargo, se consideró inviable la realización de una biopsia hepática para confirmar el diagnóstico debido a la gestación. El riesgo superaba los beneficios y se contaba con una alta probabilidad para el diagnóstico de HAI. Una limitación de este reporte de caso fue la ausencia de confirmación por biopsia hepática. Sin embargo, la respuesta favorable a la corticoterapia en nuestra paciente confirma la hipótesis de que la presentación fue atribuible a una respuesta autoinmune desencadenada por la infección por el virus del dengue, que llevó a insuficiencia hepática aguda. Esta ocurrencia es rara debido a los cambios protectores inmunológicos durante el embarazo. Los niveles elevados de estrógeno y progesterona contribuyen a un aumento de las células T reguladoras y Th2, involucradas en la vía antiinflamatoria, mientras que disminuyen la expresión de las células Th1 y Th17, correspondientes a la vía proinflamatoria ^17^. La corticoterapia resultó efectiva y se logró retirar a la paciente de la lista de trasplante hepático. Durante el período posparto, se introdujo la azatioprina junto con la terapia continua con prednisona para mantener el control de la enfermedad, dado el alto riesgo de brotes debido a la disminución de los niveles hormonales, que restablecen el estado proinflamatorio de las células Th1 y Th17 ^18^. El monitoreo cercano durante los primeros seis meses posparto es crucial para identificar brotes de la enfermedad ^12^^,^^17^^,^^19^.

La respuesta positiva a la corticoterapia confirmó nuestra sospecha de HAI en una paciente infectada con el virus del dengue. Es crucial que los pacientes que responden bien inicialmente a altas dosis de corticoides sean considerados para terapia adyuvante, como la azatioprina, mientras se reduce gradualmente la dosis de corticoides para minimizar los efectos secundarios y mantener una buena respuesta clínica y de laboratorio ^12^^,^^17^. El seguimiento regular es esencial durante al menos un par de años después del diagnóstico para mantener el control de la enfermedad y prevenir recaídas ^17^.

Presentamos el primer caso de una gestante con infección por el virus del dengue que posteriormente desarrolló HAI, confirmada por el curso clínico, marcadores inmunológicos y respuesta positiva a la corticoterapia y la azatioprina. Es importante que las regiones con alta prevalencia de infección por dengue y pacientes susceptibles a enfermedades autoinmunes consideren esto como un potencial diagnóstico diferencial. El mecanismo exacto por el cual este virus desencadena la HAI en pacientes susceptibles sigue siendo incierto, por lo que se necesitan más estudios, debido a la alta prevalencia de enfermedades transmitidas por mosquitos a nivel mundial.
